# Drone routing problem model for last-mile delivery using the public transportation capacity as moving charging stations

**DOI:** 10.1038/s41598-022-10408-4

**Published:** 2022-04-15

**Authors:** Amirhossein Moadab, Fatemeh Farajzadeh, Omid Fatahi Valilai

**Affiliations:** 1 Department of Finance and Management Science, Carson College of Business, Washington State University, Pullman, WA USA; 2 Data Science, Worcester Polytechnic Institute, Worcester, MA USA; 3 Department of Mathematics and Logistics, Jacobs University Bremen, Bremen, Germany

**Keywords:** Electrical and electronic engineering, Mechanical engineering

## Abstract

The fast and cost-efficient delivery of goods ordered online is logistically a challenging problem. Many firms are looking for ways to cut delivery times and costs by exploring opportunities to take advantage of drone technology. Deploying drones as a promising technology is more efficient from both environmental and economic perspectives in last-mile delivery. This paper considers a last-mile delivery system in which a set of drones are operated in coordination with public transportation system to deliver a set of orders to customer locations. A mathematical model based on Vehicle routing Problem (VRP) is extended to solve this problem. A real-world case inspired by Bremen 2025 transportation paradigm is also developed to validate the developed mathematical formulation. Results show that the sequence of visiting customers and public transport stations highly impacts the remaining charge and efficiency of drone tour planning. Also, using public transport vehicles, which enables drones to charge their battery or to approach customers, can reduce the number of drones required for satisfying the demands in a service area. The results show that there are high potentials to save energy for drone-enabled last-mile delivery by using the public transportation network.

## Introduction

Last-mile delivery refers to the final transportation of goods from distribution centers toward customers, which is often considered as the most expensive and the most complicated process of a supply chain^1,2^. For many companies, the so-called last-mile distribution to the customers can account for up to 28% of the total transportation cost^3^. Similarly, it is reported that the total cost of global parcel delivery operations will exceed 70 billion Euros a year, with last-mile delivery constituting more than 50% of these total costs^4^. From an operational perspective, optimal planning of last-mile delivery routes constitutes a particularly challenging and costly problem because of two main global changes: the rise of e-commerce and rapid global urbanization^5^.

With the boom of e-commerce, consumers are expecting increasingly fast and responsive delivery services, which shifted market share from business-to-business (B2B) to business-to-consumer (B2C). On the other hand, the continued growth of traffic loads on roads due to global urbanization created major obstacles to the successful delivery of goods and services to customers. The difficulties associated with urban distribution systems made the conventional delivery vehicles an unsatisfactory means of transportation for goods delivery due to their high fuel costs, delays in populated metropolises, and the impact on the environment^6^. In urban areas, the predominant factor for productivity of a distribution system is to reduce the negative impact of travel time, and, in turn, increase the service level^7^. Therefore, increasing number of city Logistic Service Providers (LSPs) which are experimenting with alternative vehicle technologies to overcome the impending limitations to the efficiency of conventional delivery operations.

Moreover, according to the seventeen interconnected Sustainable Development Goals (SDGs) proposed in the united nations 2030 agenda, there is an urgent need to take these goals into the decision to achieve a better and more sustainable future^8^. So, as LSPs are trying to keep their delivery times and costs at a minimum level, they also must think about providing a solution which reduces the negative environmental impacts last-mile distribution to address the goal number 11 and 13 of SDGs. Most of these efforts are focused on the integration of solutions motivated by sustainable cities and communities^9^. Nowadays, a city LSP should develop distribution strategy able to orient delivery operations by considering both economic and environmental aspects.

The drone has been found as a promising development that can improve the “last-mile” delivery of products to consumers, both from an economic and environmental perspective. First, from an economic perspective, drones are not restricted to a discrete set of static roadways, and they can move flexibly in three dimensions. This capability allows them to circumvent traffic congestion or accidents and, as a result, traveling at more constant and higher average speeds, which can reduce delivery times substantially. Second, drones exhibit a very low Greenhouse Gas (GHG) and pollutant emissions footprint from an environmental perspective because of being powered by electric engines with rechargeable batteries^10^ presented a novel framework to analyze the real-world energy and emissions efficiency of drones. The proposed framework proved that drones are more efficient in terms of CO2 emission (around 47 times) and energy consumption than typical diesel delivery. These most obvious advantages of deploying drones over conventional vehicles make them an appealing solution to enable sustainable distribution systems by reducing GHG emitted and reducing traffic congestion^11–13^. Although drone delivery offers a more efficient and more environmentally friendly alternative to traditional truck delivery, it should be noted that there are two major limitations in terms of the flight endurance of the drone’s battery and the capacity that these flying vehicles can carry.

These two drawbacks impede the possibility of using only drones as an alternative to traditional truck delivery in last-mile logistics. To increase the flight range of drones, which is restricted by its limited battery capacity, most attempts by companies and research studies focus on using a truck-drone hybrid delivery system^14–16^. Unlike prior research, this study aims to use public transportations as mobile charging stations to extend drones' flight range. The idea of using public transport networks such as buses as an innovative and novel way to increase the drones' travel range is firstly introduced by Stanford researchers reported in^17^. They designed a comprehensive algorithmic framework for solving the problem of multi-drone goods delivery by considering public transport bus services as a solution to save drones' energy. Generally, using public transportations (e.g. commuter or public buses, tram) instead of trucks to increase the drone's flight range has two main advantages. First, the public transportation network typically has its separate route that is not affected by urban traffic. So, they are more efficient in terms of delivery time and energy consumption. Second, public transportation services are the already built infrastructure and their existence is necessary to commute in urban area. Therefore, utilizing them instead of building new infrastructure, such as charging stations proposed in smart city conceptual framework^18,19^ or using other vehicles, such as trucks, can significantly cut operational costs and reduce greenhouse gas pollution.

**Figure 1:**
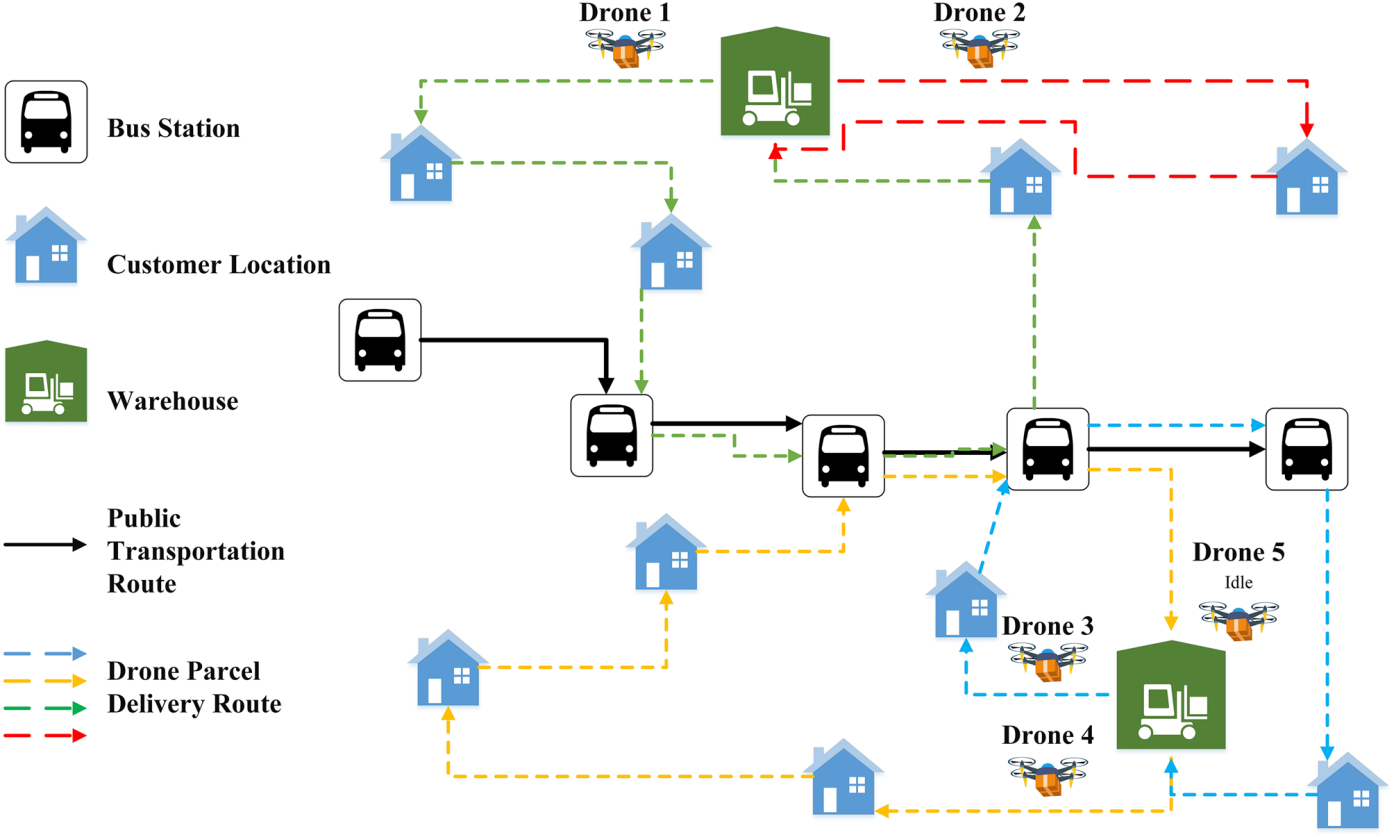
A general depiction of the proposed drone delivery system.

This paper deals with the problem of delivering low-weight orders to a set of customer locations using multiple drones that operate in conjunction with public transportations. Therefore, it allows drones to deliver packages more efficiently, which the majority of them are within the payload capacity. For instance, 86% of orders shipped by Amazon weigh less than 3 kg (6.5 lb)^20^, which is less than the common drones' weight range^21^. This work is the first to develop an optimization model for delivering packages across broad urban areas with multiple drones, lunching from multiple depots, and using multiple public transport vehicles as a moving charging station. The proposed model assumed that drones could deliver one or multiple packages to customer locations per trip. Meanwhile, drones can also save the energy and charge their battery by hopping on public transport if the energy required for completing the assigned route is not enough. Note that the main purpose is to consume minimum energy, so drones tend to use public transportation as much as possible to serve customers and complete delivery operations. The conceptual model of the defined problem is illustrated in Fig. 1.

The paper is organized as follows. Second section reviews the literature on the drone delivery routing problem and states the contribution of the current study. Third section describes the proposed problem in detail, as well as the assumptions made to formulate the problem. Fourth section presents a real case scenario for validating and implementing the developed mathematical model. It also determines the impact of input parameters on the outcomes via performing a sensitivity analysis. Fifth section discusses the practical and managerial viewpoint of the proposed approach. Finally, sixth section concludes the paper and presents directions for future research.

## Literature review

In recent years, there is a growing literature study on the potential application of drones^21^ provides an extensive overview of civil applications of drones and surveyed the literature on relevant optimization approaches. This review study reported that drones' operational planning as a means for delivery purposes is a well-studied problem in the literature. The core idea of most studies on the utilization of drone delivery is to develop an accurate model for optimizing the drones' routing and efficiently scheduling the operations^22^. Besides researchers' attention, drone technology as an innovative way of on-demand delivery has attracted the interest and investment of many reputable companies, including UPS, Amazon, Google, and DHL^23^. The performance of the drone delivery system suffers from two major technical limitations: limited battery capacity, which has an impact on the drone's flight duration, and limited payload, which means that a drone can visit a small number of customers per trip^24^. Another problem for the parcel delivery with drones is considering only drones as a transportation fleet, in such a way that drones are entirely handling the delivery operations. The pioneers in studying the truck-drone problem were^25^, who developed an optimization model with the objective function of minimizing the drone route's completion time. In this case, it is assumed that a drone can travel on a truck to serve a single customer every time the truck stops at a customer location and returns to the truck at one of its subsequent customers’ location visiting. In fact, trucks are using as mobile hubs that dispatch and receive drones along their own delivery route in this collaborative system. Recently, researchers^16^ have addressed the problem of truck-drone routing, in which, unlike their former study, customer parcels are delivered via multiple heterogeneous drones. In this problem, drones can be launched multiple times from different truck locations. The truck can also deliver customer packages between drone launch and retrieval locations while drones are airborne. Other researchers have described nearly identical problems. For example, a truck-multi-drone unified delivery system is designed by researchers^5^. Contrary to the previous research that assumed trucks have their own routs to deliver parcels, this study considered that trucks should stop at discrete customer locations along its route and launch one or multiple drones to serve other customers. The truck will wait at its current location until all drones come back and then carry drones to the next customer node as a new stop location. Researchers^15^ used constraint programming approach to address multiple trucks, multiple drones, and multiple depots scheduling and routing problems. This study extended the problem by considering two different types of drone tasks: drop and pickup. The use of multiple trucks and multiple drones is also considered by researchers^26^, in which drones fly from delivery trucks, make deliveries, and return to any available delivery truck nearby.

Another problem for the parcel delivery with drones is considering only drones as a transportation fleet, in such a way that drones are entirely handling the delivery operations. Studies on drone-only delivery systems assume multiple drones and that each drone can cover one or several customers per trip. researchers^27^ formulated and optimized the drone fleet size by using the relationship between payload, battery capacity, and flight range and minimizing the delivery system's total costs. Researchers^23^ proposed two multi-trip drone routing that drones are supposed to start their route from the single central depot and visit multiple customers per each route. To find the optimal solution of the proposed system, the first objective function minimizes the total operating cost subject to a delivery time limit, and the second one minimizes delivery time subject to a budget constraint. Researchers^28^ developed a mathematical formulation to find the optimal routing of multiple drones, starting their routes from multiple depots. Because of drones’ limited battery endurance, the possibility of recharging drones' batteries during their routes is considered in the presented model. Similarly, a multi-trip drone routing delivery system with time windows is designed by researchers^29^.

Recently, some research has been done investigating the use of public transportation networks in drone parcel delivery to enlarge the range of flying and services^30,31^. Some characteristics, such as simultaneously considering the capability of charging batteries on the top of public transport vehicles, considering multiple deliveries per drone trip, and multiple warehouses, distinguish the current study from similar papers in drone parcel delivery using public transport. A summary of studies published in the context of the drone delivery system is gathered in Table 1. The practical assumptions of the current study are highlighted further in the last row of the table.

**Table 1:** Summary of papers on drone delivery routing problem.

Research	Type of fleets		Objective(s)	Problem				Solution approach
	Drone	Truck and Drone		#Drones	#Depots	#Cust/trip	Charging method	
^32^	✓		Min delivery lateness	multiple	multiple	1	Replacing batteries at depots	Heuristic
^33^		✓	Min time	1	1	multiple	Replacing battery at depot	Route split
^34^		✓	Min route completion time	multiple	1	multiple	Recharging batteries by using truck	Heuristic
^16^		✓	Min latest return time to depot	multiple	1	1	Saving energy by using trucks	Heuristic
^24^	✓		Max covered distance and tasks	multiple	multiple	multiple	Recharging batteries at depots	Receding horizon task assignment
^35^		✓	Min time	1	1	1	Saving energy by using truck	Dynamic programming
^14^		✓	Min waiting time	multiple	1	1	Saving energy by using truck	Meta-heuristic
^23^	✓		Min time and cost	multiple	1	multiple	Replacing batteries at depot	Simulated annealing
^29^	✓		Min cost and energy	multiple	1	multiple	Replacing batteries at depot	Branch-and-cut
^36^		✓	Min return time to depot	1	1	1	Recharging battery by using truck	Branch-and-cut
^15^		✓	Min max completion time	multiple	multiple	multiple	Saving energy by using truck	Constraint programming
^37^		✓	Min return time to depot	1	1	multiple	Replacing battery by using truck	Simulated annealing
This study	✓		Min energy	multiple	multiple	multiple	Recharging batteries by using public transport	Exact

^#^ cust/trip: number of customers per drone trip.

### Benefits of using public transport

There is an exploding body of literature on potential application scenarios of the drones-truck hybrid delivery systems in recent years^5^. The limited delivery capacity issues and relatively short travel distance because of low energy capacity level are caused scholars to combine ground vehicles in covering a service area^14,37^. This section summarizes the benefits of using public transport instead of other alternatives such as trucks to overcome drone operational restrictions and further looks into other planning aspects that must be incorporated in the business planning approaches in harmony with the 2030 sustainable blueprint^8,38^. The European Commission classifies all means of transportation as a green vehicle when the emissions intensity does not exceed 1.2 kg of CO2 emissions per km, considering the potential in decarbonizing, reducing air pollution, and increasing system efficiency^39^. The introduction of green vehicles requires adaptation in last-mile distribution systems due to its relevant impact on the e-commerce market and the rise of on-demand logistics. A group of researchers focused on green vehicles' challenges and opportunities, including drones, to discuss the urban planning approaches in the development and implementation phase^40^. The impact of drones on CO2 emission and delivery cost is investigated using a routing model to simulate real-world scenarios and exploit sustainability aspects^41^. This study proved that using only drones can save variable costs, primarily the expenditure on fuel, which subsequently dramatically reduces carbon emissions. Although unimodal use of drones demonstrated their efficiency from multiple planning aspects like including environmental, delivery time, and costs in urban distribution systems^39,42^, multimodal delivery systems can provide additional cost savings and carbon footprints reduction. Multimodal delivery systems increase the possibility of boosting drone battery life in energy storage and expanding the service area. However, multimodality must also consider urban congestion, not an additional environmental burden and faster delivery expectations. To avoid these issues, adapting the existing city facilities such as rooftops of city buildings^43^ and public transports^44,45^ are novel ways to improve urban delivery systems. These studies showed that using already built city facilities to address drone operational challenges is beneficial from a customer, business, environmental aspects. Customers will have faster delivery because of not stuck in traffic (most public transports are operated on established routes which are separated from the primary traffic such as Germany, Singapore, China), businesses are not required to spend additional investment on buying or building facilities for drones battery charges, and the environment will be safe from extra emissions as a result of using other fuel-based vehicles as well as the opportunity of extending drones battery life. Moreover, due to the wide coverage of public transportation in residental areas, the proposed idea in this paper will be easily expandable and scaleable to fulfill the customer orders with agility.

## The multi-depot drone routing problem

### Problem description

**Figure 2:**
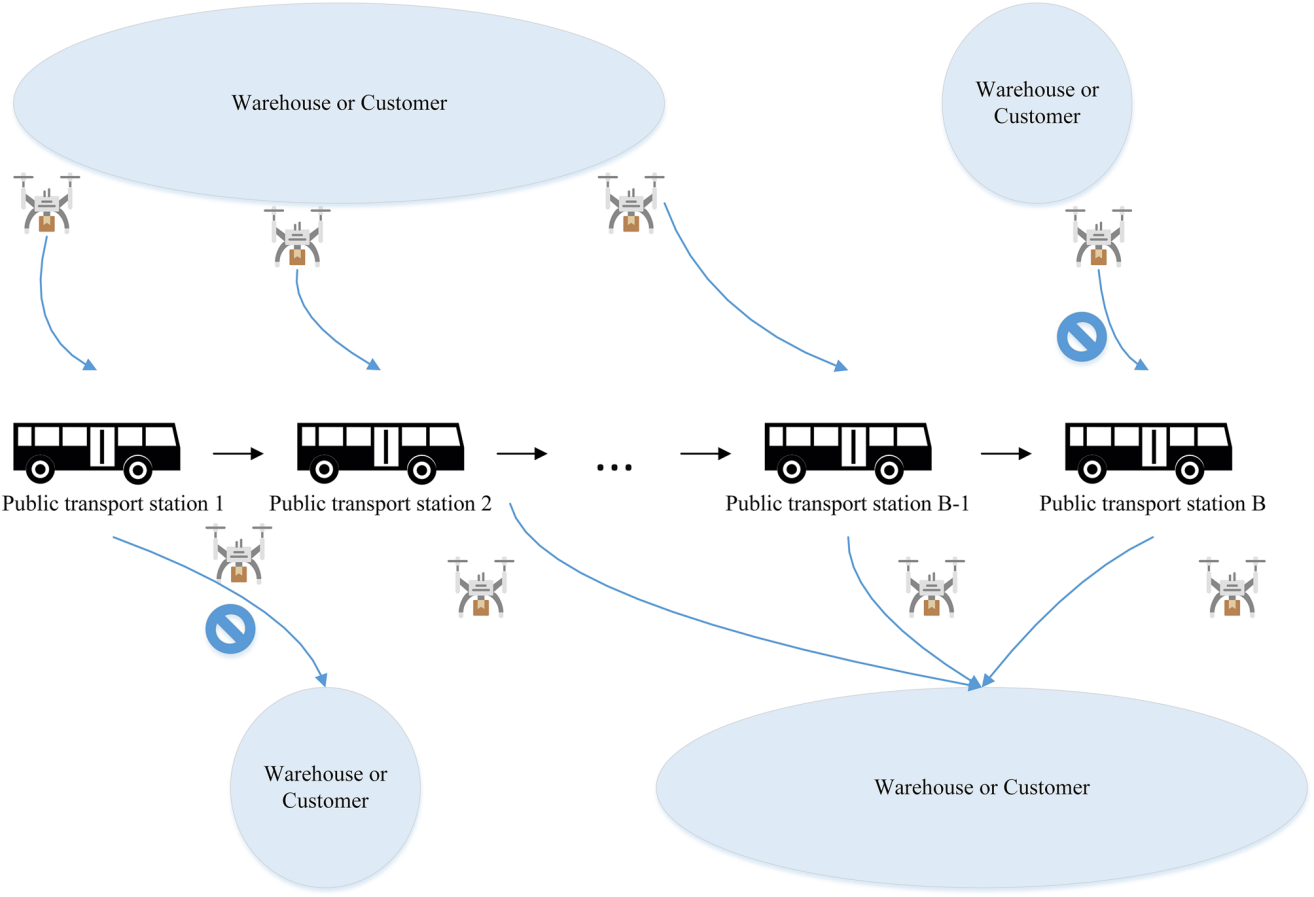
Merging procedure at public transport stations.

This section describes the main assumptions of the defined problem and presents the associated mathematical model. The objective function of the problem aims at minimizing the total required energy for the last-mile delivery of customer orders. Hence, the following key decisions must be optimized. (1) assignment of customer locations to depots, (2) assignment of customers' packages to drones, (3) sequence of visiting customer locations and public transport stations, and (4) assignment of flying drones to pre-existing public transport routes, if required. In this problem, not only do drones use public transportation for charging purposes, but also they may use them for traveling between locations without further consuming energy. The presumed procedure for using public transport is illustrated in Fig. 2. Drones can only be mounted on public transport at the first (public station 1) and middle stations for charging operations, either from a warehouse node or customer node. Unlike station 1, drones are not allowed to hop on public transport at the last station (station |B|) due to the relatively long suspension of public transport operation until its resumption. So, drones can only leave station |B| to travel toward a warehouse or customer node and are not allowed to enter the last station. Also, drones are not allowed to leave the first station toward a warehouse or customer node because the station is the start point, and the public transport has not yet resumed its operation, which as a result, the drone battery did not receive energy.

The following additional assumptions are considered in the problem:

- Multiple depots, multiple drones, and a single public transport route are assumed.
- Drones start and end their route from the same depot.
- All drones are homogenous in shape, capacity, and speed.
- The public transport route is considered to have only a forward direction.
- Each customer location must be visited just once, and its order should be fulfilled in a single drone visit.
- The energy used by the drone depends on its travel speed. Accordingly, energy use per distance traveled is measured as a function of power consumption.
- As far as the capacity of drones does not exceed, they can serve multiple customers per trip.
- The distance between each pair of nodes is symmetric.
- The distance between all the stations is the same. And, drones use public transport to recharge their battery or save their energy.
- The drone’s waiting time in bus station to join and arising from the coming bus has been assumed to be neglectable. Also, it can be assumed that in each us station a waiting infrastructure for drones can be assumed.
- During the traveling of drones on top of public transportations, energy consumption is considered zero.
- Any warehouse can fulfill the demand of customers. In other words, warehouses are similar in type and number of products, and their inventory will not face a shortage.
- Drones can only get on and take off public transportation vehicles at the stations, not between stations.

### Mathematical formulation

Three following main sets of constraints and notations will be used to formulate the problem mathematically.

- Route feasibility and synchronization constraints
- Capacity and weight tracking constraints
- Energy tracking and charging policy constraints

Sets

I, J, L: Set of all nodes including warehouses, customers, and public transport stations(i, j, l = 1, …, |I|)
K: Set of drones (k = 1, …, |K|)
B: Set of public transport stations (b = 1, … ,|B|)
Wa: Set of warehouses (wa = 1, …, |Wa|)
C: Set of customers (c = 1, …, |C|)

Parameters

υ: The maximum drone’s speed
E: The maximum energy can be stored in the drone’s battery
Q: The maximum weight capacity of the drone
N: Number of available drones at each depot (warehouse)
α: The energy conversion efficient
S: The number of stations needed to be traveled by drones to charge their batteries
M: A large constant
*ε*: A small constant
d_j_: Weight of customers demand at location j∈C
dis_ij_: Distance between node i and node j

Decision variables

$$x_{ij}^{k}$$
Binary variable equal to 1 if drone k ∈ K traverses the arc between node i ∈ I and j ∈ I, 0 otherwise.
$$a^{k}$$
Binary variable equal to 1 if drone k ∈ K is used, and 0 otherwise.
$$v^{k}$$
The total weight that drone k ∈ K picks up for delivery operations.
$$w_{i}^{k}$$
Weight of drone k ∈ K at location i ∈ I.
$$r_{i}^{k}$$
Remained energy of drone k ∈ K at location i ∈ I.
$$\psi_{j}^{k}$$
The auxiliary variable of remained energy for linearization of drone k ∈ K at station j ∈ B.
$$u_{i}^{k}$$
Positive variable considered in sub tour elimination constraint.

Objective function

1 $$ {\text{Minimize}}\quad \sum\limits_{i \in Wa,C} {\sum\limits_{j \in J} {\sum\limits_{k \in K} {\alpha \left( {\frac{{dis_{ij} }}{\nu }} \right)x_{ij}^{k} } } } + \sum\limits_{i \in B} {\sum\limits_{j \in Wa,C} {\sum\limits_{k \in K} {\alpha \left( {\frac{{dis_{ij} }}{\nu }} \right)x_{ij}^{k} } } } $$

The objective function (1) aims to minimize the total energy that drones consumed in delivery operations. As drone traveling over the bus does not endure energy consumption to drones, it is not included in the objective function.

Subject to:

(A) Route feasibility and synchronization

2 $$ \sum\limits_{i \in I} {\sum\limits_{k \in K} {x_{ij}^{k} } } = 1\quad \forall j \in C $$

3 $$ \sum\limits_{i \in Wa} {\sum\limits_{j \in C \cup B} {x_{ij}^{k} } } \le 1\quad \forall k \in K $$

4 $$ \sum\limits_{j \in J} {x_{ij}^{k} } \le 1\quad \forall i \in Wa,\forall k \in K $$

5 $$ \sum\limits_{j \in C \cup B} {\sum\limits_{k \in K} {x_{ij}^{k} } } \le N\quad \forall i \in Wa $$

6 $$ Ma^{k} \ge \sum\limits_{i \in I} {\sum\limits_{j \in C \cup B} {x_{ij}^{k} } } \quad \forall k \in K $$

7 $$ \varepsilon a^{k} \le \sum\limits_{i \in I} {\sum\limits_{j \in C \cup B} {x_{ij}^{k} } } \quad \forall k \in K $$

8 $$ \sum\limits_{i \in I} {x_{ij}^{k} } = \sum\limits_{i \in I} {x_{ji}^{k} } \quad \forall j \in I,\forall k \in K $$

9 $$ \sum\limits_{j \in I} {x_{ij}^{k} } = \sum\limits_{j \in I} {x_{ji}^{k} } \quad \forall i \in Wa,\forall k \in K $$

10 $$ x_{j(j + 1)}^{k} + \sum\limits_{i \in Wa \cup C} {x_{ji}^{k} } = x_{(j - 1)j}^{k} + \sum\limits_{i \in Wa \cup C} {x_{ij}^{k} } \quad \forall j \in B\backslash \{ 1,\left| B \right|\} ,\forall k \in K $$

11 $$ \sum\limits_{i \in Wa \cup C} {x_{ij}^{k} } = x_{j(j + 1)}^{k} \quad \forall j \in B\backslash \{ 2,...,\left| B \right|\} ,\forall k \in K $$

12 $$ x_{(j - 1)j}^{k} = \sum\limits_{i \in Wa \cup C} {x_{ji}^{k} } \quad \forall j \in B\backslash \{ 1,...,\left| B \right| - 1\} ,\forall k \in K $$

13 $$ \sum\limits_{i \in Wa \cup C} {x_{ij}^{k} } + x_{(j - 1)j}^{k} \le 1\quad \forall j \in B\backslash \{ 1\} ,\forall k \in K $$

14 $$ x_{ij}^{k} = 0\quad \forall i,j \in B:j \ne i + 1,\forall k \in K $$

15 $$ x_{ij}^{k} + x_{jl}^{k} \le 1\quad \forall i,l \notin B,\forall j \in B,\forall k \in K $$

16 $$ u_{i}^{k} - u_{j}^{k} + |I|x_{ij}^{k} \le |I| - 1\quad \forall i,j \in B \cup C:i \ne j,\forall k \in K $$

Constraint (2) ensures that each customer is visited only once. Constraint (3) makes sure that each drone can be assigned to at most one warehouse. Constraint (4) guarantees that each assigned drone can only fly toward one location, either customer or public transport station. Constraint (5) shows that each warehouse has a limited number of available drones for delivery operation. Constraints (6) and (7) indicate that if a drone has been selected for delivery, it should visit at least one customer. The flow conservation between nodes is incorporated in constraints (8) and (9). Constraints (10)–(15) are set to track the public transportation route as shown in Fig. 2. Constraint (10) shows the flow between the public transportation stations, excluding the first and last stations in which fits the symmetry between entrance arcs which origins from a warehouse, customer, or a previous bus station, and exit arcs which destine to another location including customer, or a warehouse, or next station. The flow of the first and the last station are indicated in constraints (11) and (12). As there is no previous station for the first station, the only entrance arc would be from a warehouse or a customer. Besides, drones are assumed to use public transportation for traveling and charging batteries, so bus stations are not used as hubs, and there is only one exit arc destines to the next bus station.

According to what is assumed in the merging procedure shown in Fig. 2, constraint (13) shows that for all bus stations except the first station, each drone can be on the top of public transport under at most one of three situations: taking public transport from the customer nodes, taking public transport from the warehouse nodes, continuing the delivery route from the previous station. And constraint (14) ensures the continuity of the public transport route according to the order of the stations. Constraint (15) prevents drones from using bus stations as a hub or a simple middle nod since the goal of applying public transportation in parcel delivery differs. Sub-tour elimination constraints are considered in constraint (16).

(B) Capacity and weight tracking

17 $$ \sum\limits_{i \in I} {\sum\limits_{j \in C} {d_{j} x_{ij}^{k} } } \le Q\quad \forall k \in K $$

18 $$ \sum\limits_{i \in I} {\sum\limits_{j \in C} {d_{j} x_{ij}^{k} } } {\text{ = v}}^{k} \quad \forall k \in K $$

19 $$ w_{i}^{k} \ge v^{k} - M\left( {1 - \sum\limits_{j \in B \cup C} {x_{ij}^{k} } } \right)\quad \forall i \in Wa,\forall k \in K $$

20 $$ w_{i}^{k} \le v^{k} + M\left( {1 - \sum\limits_{j \in B \cup C} {x_{ij}^{k} } } \right)\quad \forall i \in Wa,\forall k \in K $$

21 $$ w_{j}^{k} \ge w_{i}^{k} - d_{j} - M\left( {1 - x_{ij}^{k} } \right)\quad \forall i \in I,\forall j \in B \cup C,\forall k \in K $$

22 $$ w_{j}^{k} \le w_{i}^{k} - d_{j} + M\left( {1 - x_{ij}^{k} } \right)\quad \forall i \in I,\forall j \in B \cup C,\forall k \in K $$

Constraint (17) incorporates the limited weight capacity of drones, while constraints (18)–(22) update the pick-up and delivery load of the drone at each type of node. Constraint (18) indicates the total loaded packages that each drone is supposed to deliver. Constraints (19) and (20) define each drone's weight in the location of the warehouses as the amount of demands weight assigned to the drone. Finally, each drone's carrying weight in the stations and customers' location is updated in constraints (21) and (22) based on the delivered demand at each node. Note that demands' weight is only defined in the customers' locations and is zero at stations.

(C) Energy tracking and charging policy

23 $$ r_{i}^{k} - \sum\limits_{j \in I} {\alpha \left( {\frac{{dis_{ij} }}{\nu }} \right)x_{ij}^{k} } \ge 0\quad \forall i \in Wa \cup C,\forall k \in K $$

24 $$ r_{i}^{k} - \sum\limits_{j \in Wa \cup C} {\alpha \left( {\frac{{dis_{ij} }}{\nu }} \right)x_{ij}^{k} } \ge 0\quad \forall i \in B,\forall k \in K $$

25 $$ r_{i}^{k} = {\text{E}}\sum\limits_{j \in I} {x_{ij}^{k} } \quad \forall i \in Wa,\forall k \in K $$

26 $$ r_{j}^{k} \le r_{i}^{k} - \alpha \left( {\frac{{dis_{ij} }}{\nu }} \right) + M\left( {1 - x_{ij}^{k} } \right)\quad \forall i \in Wa \cup C,\forall j \in B,\forall k \in K $$

27 $$ r_{j}^{k} \ge r_{i}^{k} - \alpha \left( {\frac{{dis_{ij} }}{\nu }} \right) - M\left( {1 - x_{ij}^{k} } \right)\quad \forall i \in Wa \cup C,\forall j \in B,\forall k \in K $$

28 $$ r_{j}^{k} \le \psi_{j}^{k} + M\left( {1 - x_{(j - 1)j}^{k} } \right)\quad \forall j \in B\backslash \{ 1\} ,\forall k \in K $$

29 $$ r_{j}^{k} \ge \psi_{j}^{k} - M\left( {1 - x_{(j - 1)j}^{k} } \right)\quad \forall j \in B\backslash \{ 1\} ,\forall k \in K $$

30 $$ \psi_{j}^{k} \le \frac{{\text{E}}}{S} + r_{j - 1}^{k} \quad \forall j \in B\backslash \{ 1\} ,\forall k \in K $$

31 $$ \psi_{j}^{k} \ge r_{j - 1}^{k} \quad \forall j \in B\backslash \{ 1\} ,\forall k \in K $$

32 $$ \psi_{j}^{k} \le E\quad \forall j \in B\backslash \{ 1\} ,\forall k \in K $$

33 $$ r_{j}^{k} \ge r_{i}^{k} - \alpha \left( {\frac{{dis_{ij} }}{\nu }} \right) - M\left( {1 - x_{ij}^{k} } \right)\quad \forall i \in I,\forall j \in C,\forall k \in K $$

34 $$ r_{j}^{k} \le r_{i}^{k} - \alpha \left( {\frac{{dis_{ij} }}{\nu }} \right) + M\left( {1 - x_{ij}^{k} } \right)\quad \forall i \in I,\forall j \in C,\forall k \in K $$

35 $$ v^{k} ,w_{i}^{k} ,r_{i}^{k} ,u_{i}^{k} ,\psi_{j}^{k} \ge 0\quad \forall i \in I,\forall k \in K $$

36 $$ x_{ij}^{k} ,a^{k} \in \left\{ {0,1} \right\}\quad \forall i,j \in I,\forall k \in K $$

Constraint (23) and (24) ensure that each drone can fly from one node to another if its remaining energy is enough for traveling the rest of the assigned route. Constraint (25) mandates that each drone starts its tour from a warehouse with a fully charged battery. The remaining energy of drones at stations is defined in constraints (26)–(32). Constraints (26) and (27) update drones' remaining energy at each station if drones start using public transport from customer or warehouse location. Although the energy consumed by the drones is based on a factor of the required flying time between locations, this energy consumption function is equal to zero when drones take public transportation. So, drones' remaining energy at these stations, excluding the first station, is updated by defining an auxiliary variable in constraints (28) and (29). According to this model's assumptions, drones need to travel by public transport and pass S number of stations to be fully charged. In this regard, constraints (30) and (31) present the remained energy at each station, which will be equal to the sum of the amount of energy available from the previous location (r_j−1_^k^) and the energy gained through charging if public transport is used, and r_j−1_^k^ otherwise. Also, constraint (32) ensures the remaining energy after charging cannot exceed drones' energy capacity. Constraints (33) and (34) keep track of the remaining energy at each customer node. Constraints (35) and (36) specify the type of each variable used in this model.

## Numerical example and sensitivity analysis

This section examines the formulated model using a real-world scenario inspired by the Bremen 2025 transportation paradigm. In the designed case study, a subset of Bremen city public bus transportation routes is considered as moving charging stations for in-operation drones. It is assumed that all the drones can fly with a speed of 10 m/s and have the physical carrying capacity of 3 kg^39^. The maximum energy that can be stored in a drones' battery is 200 joules^46^. The drone must take a city bus for the specific number of stations to be fully charged. In this case, the traveling time between two stations is required for drones to fully charge their battery.

The alpha (α), coefficient of energy conversation is calculated based on the flight range of different types of drones used for parcel delivery^39^. According to this analytical study, drones' energy to deliver a package depends on the speed of flying, and in some kinds of drones such as Quadcopters, energy consumption per distance in the loaded drone is similar to unloaded. Since the drone's speed is considered 10 m/s, and the longest flight distance is about 4 km, the coefficient of turning time to energy is assumed to be 0.5 j/s in the presented real-world scenario hereunder. The example problems are solved using Gams software with Gurobi solver on an Intel® Core™ i3 6100 CPU @ 3.7 GHz, 8 GB RAM system.

### Case study

**Figure 3:**
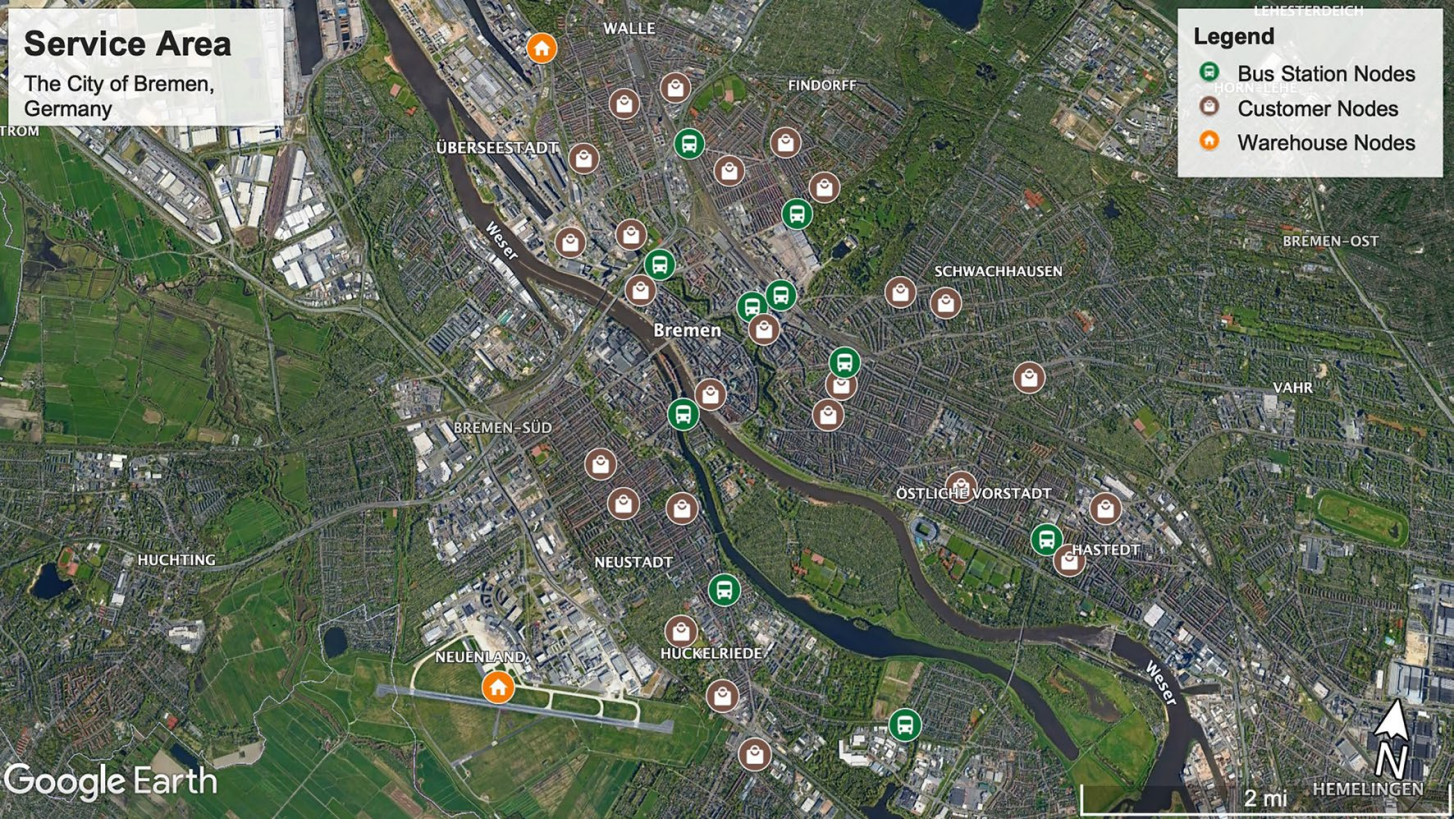
The service area representing the locations of public bus stations, warehouses, and customers in the city of Bremen, Germany, Google© Map (Aug, 2020).

Validation of this model has been verified by gathering actual parameters based on the distributed location of warehouses, customers, and public bus stations in Bremen, Germany. The service area that is used for delivery operation is illustrated in Fig. 3 and can be accessed via http://tiny.cc/BremenDroneVRP01. The distance between all types of nodes is calculated based on the coordination of locations extracted from the Google© Earth website and shared in the data repository 10.6084/m9.figshare.12888563.

**Figure 4:**
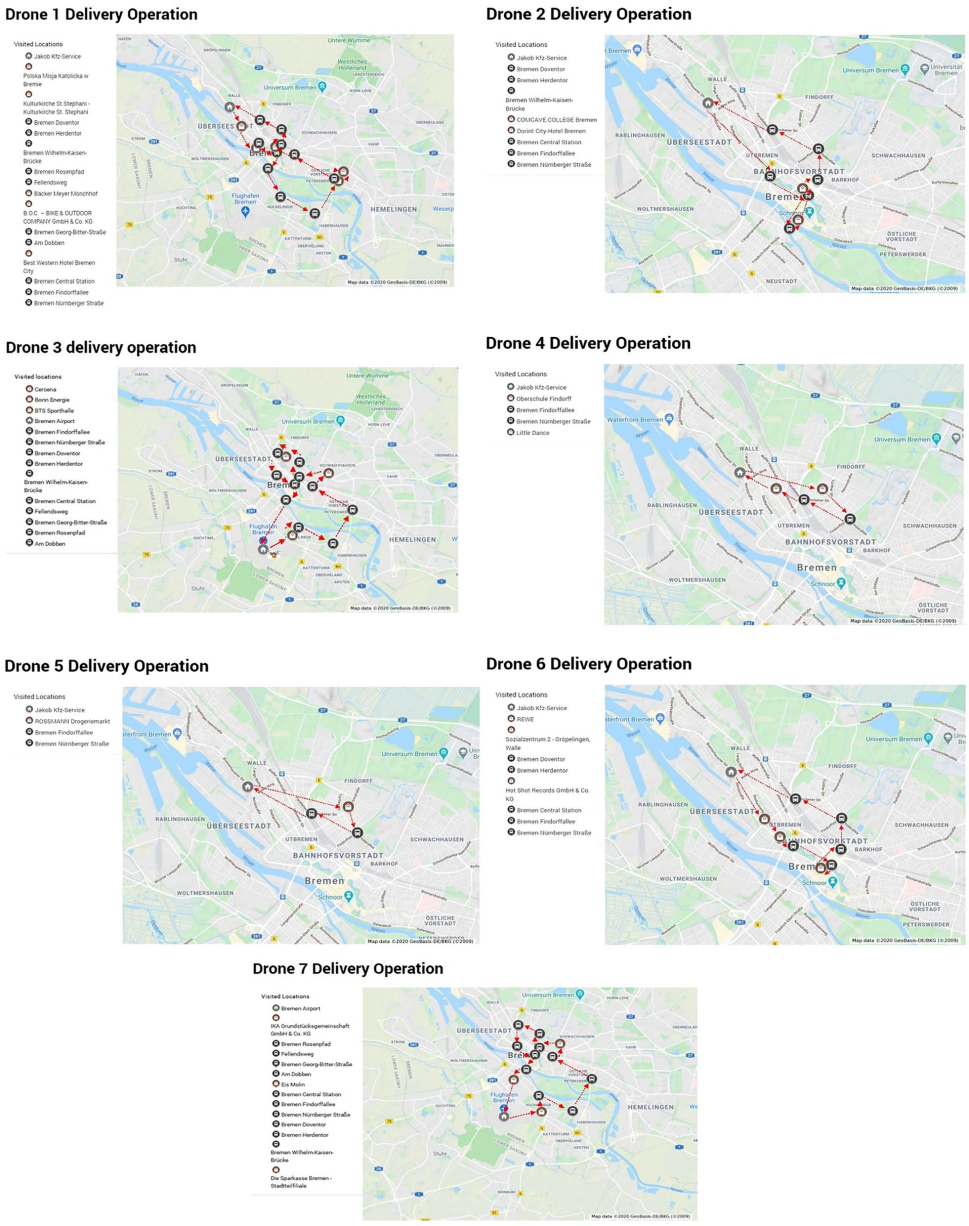
The best routing to deliver the packages.

The model has set up two warehouses as depots, each with five drones, Bremen Airport and Jakob Kfz-Service, where drones can start their delivery operations. All customers have a specific demand that is randomly assigned between 0.2 and 3 kg in this setting. Also, ten bus stations are assumed to increase the flight range of drones via battery endurance enhancement. The paper investigates the best delivery routing and assignment of orders to drones via solving the formulated Mixed Integer Linear Programming (MILP) model. Figure 4 demonstrates the best solution of the numerical experiment. As an example, drone one delivery route includes: Jakob Kfz-Service (warehouse)-Bremie (as a first customer)-Stepheni (as a second customer), then hop on the bus from station Doventor to Fellendsweg, then Monchhof and Bike Company as third and fourth customers, again hop on the bus for two stations to charge its battery and visit Best Western Hotel Bremen City, and finally takes buses for three stations to come back to the warehouse. According to the assumption, drone only needs two stations to be fully charged. Therefore, continuing the route on the bus aims to reach customers without consuming energy, which highlights another role of public transport in improving the delivery operations.

The results show that the sequence of visiting public bus stations and customers is an essential factor in minimizing the total required energy for satisfying all the demand. Also, warehouses' distribution can be a crucial input parameter since drones started operating mostly from the warehouse close to the service area, Jakob Kfz-Service. Nevertheless, the effectiveness of this model should be tested by examining the effect of different input parameters including warehouse locations. By changing the number of customers, in addition to the energy limit of the drones, the weight limit of the drones should also be considered. Consequently, the importance of drones number in the model must also be analyzed.

### Sensitivity analysis

To further analyze the impact of input parameters, the paper has conducted an experimental study changing the problem size in terms of the number of warehouses and customers, the warehouse locations (near to service area/far from service area), and the available number of drones at each warehouse. During experimental testing, the parameters related to drones (e.g. weight capacity, maximum energy) in this section are the same as previously described in “Case study” section. These parameters are consistent with the parameters in the studies of El-adle et al. and LBoualem et al.^39,46^.

As clearly listed in Table 2, twelve scenarios are created to investigate the impact of critical factors associated with drone last-mile delivery using public transportations. The first factor is the warehouses’ locations categorized into centralized and decentralized. The term of centralized warehouse refers to the warehouses located basically in the middle of the city and places surrounded by some probable customers. On the other hand, decentralized warehouse refers to inventory depots located outside of the town, for example, near ports, airports, or any cargo terminals. The second factor is the number of warehouses that are considered to be two or four. Besides, the importance of various factors such as the advantage of using public transportation and the significance of warehouse location can be observed by increasing customers' number in last-mile delivery. In this regard, the total number of customers is examined in three different numbers; 15, 20, and 25. Since the total number of drones under each scenario is twelve, there are six available drones for operating in scenarios with two warehouses. Similarly, this number (#ADW in the table) is three for scenarios with four warehouses. Unlike other scenarios, 16 drones considered for scenario number 11 and 12 in order to be able to solve the model with the existing system in a reasonable time (1 h).

**Table 2:** Computational result experimental test design under different scenarios.

SC.NO	Scenarios				Results								Obj
	WL	#W	#C	#ADW	#TDL W.1	#TDL W.2	#TDL W.3	#TDL W.4	#PT W.1	#PT W.2	#PT W.3	#PT W.4	Energy (Joules)
1	C	2	15	6	5	3	–	–	12	13	–	–	1526
2	C	4	15	3	3	1	3	1	8	7	6	2	1470
3	D	2	15	6	3	4	–	–	9	28	–	–	2457
4	D	4	15	3	3	2	1	3	5	8	0	11	1916
5	C	2	20	6	6	4	–	–	22	13	–	–	2061
6	C	4	20	3	3	1	2	3	8	3	4	30	1692
7	D	2	20	6	6	3	–	–	20	18	–	–	2896
8	D	4	20	3	3	2	2	3	8	5	9	9	2235
9	C	2	25	6	6	6	–	–	21	25	–	–	2320
10	C	4	25	3	3	3	3	3	3	13	8	19	1970
11	D	2	25	8	8	3	–	–	40	14	–	–	3428
12	D	4	25	4	2	1	4	4	2	10	15	36	2546

SC.NO: Scenario number, WL: Warehouse Location type, in which C stands for Centralized and D stands for Decentralized, #ADW: Number of Available Drones at each Warehouse, #TDLW.1: Number of Drones Launched from Warehouse 1, #PTW.1: Number of Public Transportation used for Warehouse 1 delivery operations, Obj: Total consumed energy, C-2–15: Abbreviated names of scenarios.

After solving the model, the best solution is summarized in the results column. Firstly, the total number of drones launched from each warehouse to fully satisfy the customers' demand (#TDL). Secondly, the total number of public transport stations used by each drones of each warehouse to complete the delivery operations in the presumed service area (#PT). And lastly, the total energy consumed by in-operation drones to deliver the packages (Obj).

According to what is summarized in Table 2, the locations of warehouse.1 is the best among others since the maximum number of drones are launched from this location for last-mile distribution. But contrary to what may be perceived, the best location is not necessarily the closest one to the service area, but the one with the shortest access to public transportation networks. This is confirmed by the fact that warehouse.1 has the most use of public transport stations. In other words, as far as the weight capacity of drones is satisfied, warehouses with the shortest distance from the public transportation infrastructure will operate their maximum drone capacity to complete the last-mile distribution. This conclusion can be examined and reliable, even in terms of energy consumption. In the scenarios with 2 warehouses, the change in the warehouses' location from the decentralized to the centralized state decreases the energy consumption in the range of 28.8% to 37.9%. This range in the scenario with four warehouses is 22.6% to 24.2%. Although drones require more energy to deliver packages in the decentralized warehouse scenario, it should also be borne in mind that operating costs in out-of-town warehouses are lower than in-town warehouses due to lower subsidies in out-of-town industrial estates. Therefore, it can be safely concluded that taking advantage of public transport infrastructure neutralizes whether the warehouses are central or decentralized. However, it is better to have warehouses outside the city and near ports to receive and store shipments at a lower cost and to reduce environmental impact of centralized warehousing.

In addition to changing warehouses' location, increasing the number of warehouses is another analyzed parameter. When the total number of warehouses increases from 2 to 4, the energy consumption decreases in the range of 3.6% to 17.8% in the centralized-warehouse scenarios and 22% to 25.7% in the decentralized-warehouse scenarios. Therefore, increasing the total number of warehouses will not cause a significant change in energy consumption thanks to the public transportation network, which has a substantial role in distributing the parcels. However, drones also have weight restrictions that will make the number of warehouses important in terms of the number of drones available for operations in each warehouse. In other words, if the number of drones available in each warehouse increases, and the warehouses are well located in terms of proximity to public transport networks, the number of warehouses will no longer matter. Thus, it is required to consider using pre-built urban transport system infrastructure along with an optimal number of drones instead of constructing more facilities such as warehouses or charging stations to completely cover the service area.

**Figure 5:**
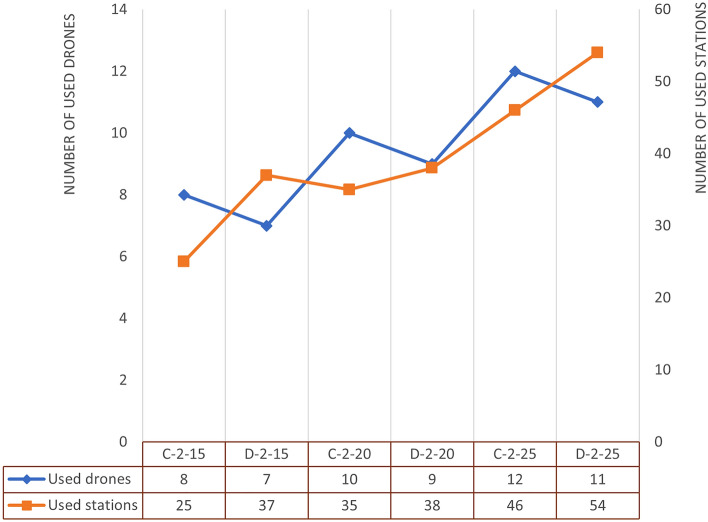
Number of stations and drones used under different scenarios with 2 warehouses.

Figure 5 highlights another role of public transportation besides its charging role for drones' battery. By changing the warehouse location from centralized to decentralized, while the number of drones used in the delivery operations decreases, the usage of public transportations increases. In decentralized warehouses, drones mostly use public transportation to access long distances for delivering packages. In this case, public transportation is used as a means of transporting drones to more remote areas. Subsequently, it is preferred to use the lower number of drones with maximum weight capacity rather than the higher number with partial weight capacity usage. Therefore, weight capacity constraints turn into binding constraints.

On the contrary, since most of the customers are in the service range of central warehouses, the role of charging is more significant than carrying drones to long-distanced destinations. Subsequently, using more drones with lower usage of weight capacity is preferable, and energy constraints are the ones that are binding constraints. These changes indicate that the model performs well under scenarios that occur in real-world instances. However, e-commerce companies and LSPs must have a systematic approach to decide on the number of drones available at each type of warehouse to achieve benefits while preserving savings.

**Figure 6:**
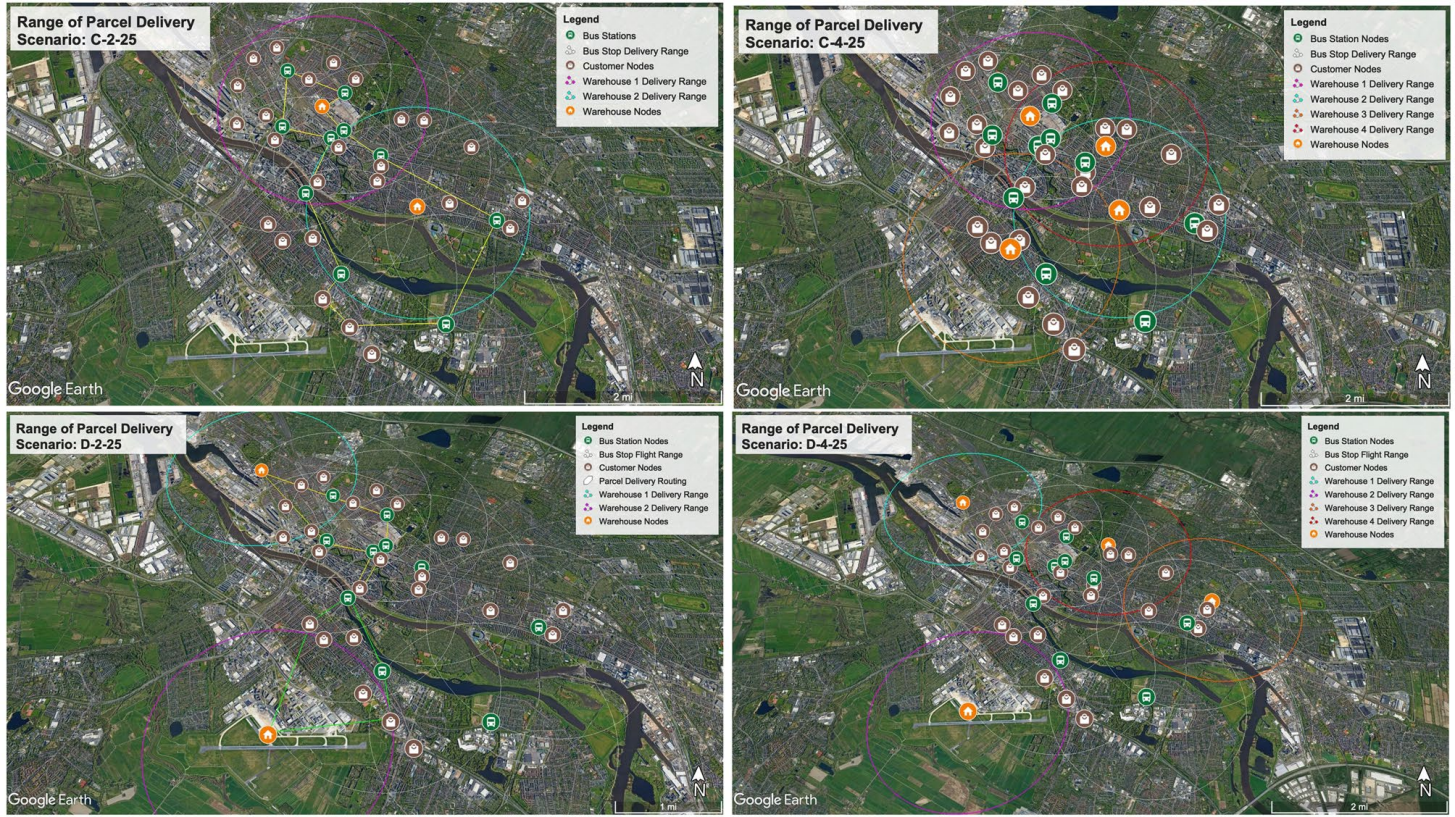
The flight range of delivery drones under four scenarios with 25 customers.

Figure 6 demonstrates the importance of using public transportation network in increasing the flight range of drones. The drones’ flight range depends on the maximum radius that can fly and return to the first place before its battery runs out. A circle with a radius of 2 km is presumed as the flight range of drones in the designed test study. As shown, each warehouse has its own 2 km coverage area, which is indicated by bold, colorful lines. For example, the area covered in 2 warehouse scenarios is depicted with pink and blue circles with a radius of 2 km. Circles with faint lines show an increase in the range covered around the warehouses, where drones will have longer battery life by using en-route recharging capability. In other words, as long as the circles around the warehouses have a common area with the circles around the public stations, drones can take advantage of the urban infrastructure, and the service level of the warehouse increases. For example, the coverage area is ~ 25 km^2^ in scenarios with two warehouses, while with the addition of faint white circles, this area increases to ~ 70 km^2^. Note that the coverage of an adequate number of customers is more important than coverage area. Therefore, the warehouses’ locations, the total number of public stations, and each station's location affect the delivery operations in terms of the total number of covered customers. For instance, in scenario C-2-25, the total number of customers located outside of the flight range of warehouse.1 and warehouse 0.2 is five, and for scenario D-2-25, this amount is 18. Increasing the number of warehouses from two to four, the total number of customers outside the flight range is one and six for scenarios C-4-25 and D-4-25. Nevertheless, connecting the public transportation network with drones will reduce the number of customers outside the warehouse’s coverage area to zero. Thus, as long as the warehouses have access to the urban transport network, they can cover areas that are even outside their radius.

## Discussion and research implications

This section summarizes the benefits of the proposed drone delivery system based on the public transportation network from three points of view: e-commerce companies and LSPs, customers, and the environment.

From a business perspective, taking advantage of emerging technologies is very important for staying competitive and retaining loyal customers. It is reported that the addressable market value of drones, uses over $127 billion by 2025^47^. Many large LSPs such as UPS and DHL and e-commerce organizations such as Amazon and Walmart have shown interest in drone delivery; here, Amazon was the first to announce an ongoing project to deploy drones for last-mile delivery^48^. The designed integrated drone-public transportation network will help companies overcome the operational restrictions of deploying drones and enhance their service levels by providing rapid parcel delivery, increasing the overall safety and efficiency of the last-mile distribution operations. Sudbury and Hutchinson^49^ analyzed that drone technology is a labor-saving/capital-using technology as drones replace labor and trucks with more specialized capital and labor in the delivery services. Also, the proposed integrated transportation network will cut the costs related to building new facilities for charging or utilizing trucks for increasing the flight range. Although drone delivery has encountered some challenges, such as legality of flying over locations, and the probability of being stolen^50^, using public transportation can solve these practical challenges.

From the customers' perspective, although the introduction of new technology has always been accompanied by resistance, public concerns about technology are mitigated over time. Currently, customers are faced with a decision between driving to a store to buy the product and waiting several days for delivery to their door. With drone delivery, the customer receives the product without the drive and just a minimal wait. They consider drone delivery a novel idea of receiving online-ordered packages^51^. This is even more appealing to customers who live on the outskirts of cities or in rural areas. This is because the coverage area increases dramatically, and drones can fly far distances using public transportation, as shown in Fig. 6. Moreover, drones can deliver packages to hard-to-reach areas, unlike trucks or traditional vehicles. All of the above will make customers more satisfied and increase the service level.

From an environmental perspective, while cars and trucks typically use gasoline or diesel fuel, drones are electric-powered. The drone’s electricity usage during delivery operation is nature friendly and has less impact on the environment. Consequently, an electric drone, charged using sustainable means, traveling to send a package is a vast improvement over a traditional vehicle on the road^52^. However, some logistics operations relevant to drones' life cycle, such as battery production and drone’s parts production, coal mining for raw material, and electricity generation, intensify global warming^53^. Experiments show that the measure of global warming indexes and the overall environmental impact in drone delivery is much less than other traditional delivery means like truck or motorcycle^54^. Companies and LSPs must also look for a balance between financial growth, environmental care, and society's health. In this sense, organizations are responsible for taking action on achieving the sustainability development goals presented in the United Nations agenda^8^. This research's optimization model can greatly reduce the greenhouse gas emissions because of connecting public transportation network to delivery operations, which results in less usage of drone’s battery and reducing the time of being in the sky.

In terms of comparison of the proposed model with previous studies, Table 3 compares the presented model from three persepecetives of success factors with highly cited pioneered papers. Moreover, as estated in the litertature^13^, drones are sustainable delivery means over traditional vehicles if they travel small distances or have a small service zone, and overall, a blended system performs best. The introduction of public transport can advantage logistics companies in both overcoming operational challenges and boosting their market with more mobile, reliable, and faster as well as less infrastructure and initial cost operation. This study is an attempt to streamline the last mile delivery process and revolutionize urban logistics. It can enable a significant improvement in service levels from a delivery time perspective. Apart from e-commerce companies and urban LSPs, for which decrease of delivery time is inevitable for competitive advantage, decrease of delivery time plays a critical and vital role in emergency and humanitarian operation situations. Recently, the importance of using drones for medical supplies deliveries has become more prominent, especially with the COVID-19 pandemics that have recently caused one of the world's biggest pandemic challenges. There are many reports that in various countries such as Canada, Germany, China, Singapore, and the UK, drones have been deployed to send supplies during quarantine^55,56^. Forbes also provides another example of how drone delivery helps the world adjust to life in emergency situations^57^ which also can be enhanced by provided idea in this paper.

**Table 3:** Impactful factors for application of Drone technology and the proposed model comparision.

	Impactful factors		
	Time and customer perception	Cost and market aspects	Energy and sustainability aspects
Research and scenarios	Highly variable on **operating** scenario^41,58^, examples: Path planning Range coverage Load balance	Highly variable on **planning** scenarios^59^, examples: Technical and operational cost Congestion cost Demand mobility Infrastructure cost	Highly variable on **logistics** scenarios^39^, examples: Network of waystations Additional warehousing Multi-modality Small batteries
^60^	Subsequent delivery in an open area with continuous traveling to minimize duration of both vehicle tandem	Limited flying range depending on refuel vehicle launch point, not applicable to land areas and costly infrastructure	Ship or airplane multi-modal with drone one or two consequent deliveries requiring additional warehousing
^25^	Minimizing total service time via drone closer to customers and large dual truck delivery with time coordination	Technical truck drivers to re-operate drones restricted to roads and customers distribution	Parallel one drone-truck delivery starting from distribution centers like Amazon warehouses
^26^	Minimizing the arrival time at the depot after delivery operation	Both trucks and drones are involved in delivering packages to customers which increases service level	Energy consumption tracking and Carbon emission of trucks are not considered
^61^	Minimization of delivery time to increase customer’s satisfaction	No need to operate new costly facilities	Mobile charging stations leads to decreasing total traversing distance which leading up to energy consumption reduction
^28^	By employing third-party delivery companies, the e-commerce company concentrates more on the quality of products and its operations, rather than delivery operation	Location of third-party logistic companies is critical, and it might impose high cost to the system	Considering factors involved in objective function and centralized location of third-party logistics led to minimization of total energy consumed during operation
Current research	Lightweight but frequent multiple parcels delivery tours starting from multiple warehouses which fulfills customer’s needs	No need to operate new costly facilities. Public route dependent delivery with energy saving possibility respecting stations distribution	Multiple drones on single public transport route with the aim of minimizing total energy on subsequent deliveries

## Conclusion and future work

The paper conducts an insightful study on drones' application in last-mile delivery, a technology-enabled efficient solution from both economic and environmental perspectives. To deal with the drone flight range limitation, this paper integrated drone delivery operations with public transportation network as a moving charging station. The primary goal of using public transport is to reduce delivery time and energy consumption of in-operation drones that may take public transportation to charge their battery and travel on public transport near to customer locations. The paper has proposed a mixed-integer linear programming optimization model for planning a set of employed drones to deliver a set of orders to customer locations per trip. The developed model is validated and implemented using a real-world scenario inspired based on the actual input parameters of nodes in Bremen, Germany. The proposed study has several future research directions. First, developing a heuristic/metaheuristic or an approximate algorithm to solve the model in a reasonable time for large scale problems. Secondly, the complex public transportation routing in big cities can be considered for developing an intelligent transport network in smart cities paradigm to encompass the drone supports for last-mile delivery especially regarding to customer order density and distribution regarding the bus station locations. Finally, the possibility of product overlaps in different warehouses and extending the proposed model to have the multi pickup possibility can be of future research interests.

## Data Availability

The service area that is used for delivery operation can be accessed via http://tiny.cc/BremenDroneVRP01. The distance between all types of nodes is calculated based on the coordination of locations extracted from the Google© Earth website and shared in the data repository https://doi.org/10.6084/m9.figshare.12888563.
